# Transplantation of human ESC-derived mesenchymal stem cell spheroids ameliorates spontaneous osteoarthritis in rhesus macaques

**DOI:** 10.7150/thno.35391

**Published:** 2019-08-21

**Authors:** Bin Jiang, Xufeng Fu, Li Yan, Shanshan Li, Dongliang Zhao, Xiaoyan Wang, Yanchao Duan, Yaping Yan, Enqin Li, Kunhua Wu, Briauna Marie Inglis, Weizhi Ji, Ren-He Xu, Wei Si

**Affiliations:** 1 Yunnan Key Laboratory of Primate Biomedical Research, Institute of Primate Translational Medicine, Kunming University of Science and Technology, Kunming, Yunnan, China; 2 Center of Reproduction, Development & Aging, and Institute of Translational Medicine, Faculty of Health Sciences, University of Macau, Taipa, Macau, China; 3 Key Laboratory of Fertility Preservation and Maintenance of Ministry of Education, Ningxia Medical University, Yinchuan, Ningxia, China; 4 Department of Radiology, the Fourth Affiliated Hospital of Harbin Medical University, Harbin, China; 5 Department of Magnetic Resonance Image, The First People's Hospital of Yunnan Province, Kunming, Yunnan, China

**Keywords:** Mesenchymal stem cells, Osteoarthritis, Spheroids, AC tranportation, Rhesus macaques.

## Abstract

It has been demonstrated that mesenchymal stem cells (MSCs) differentiated from human embryonic stem cells (hESCs), name EMSCs, can treat a variety of autoimmune and inflammatory diseases, with similar efficacies to those achieved with MSCs derived from somatic tissues such as bone marrow (BMSCs). The chance increases even higher for EMSCs, than somatic tissue derived MSCs​, to become a cell drug as the former can be produced in large scale from an unlimited hESC line with easier quality control and less biosafety concern. We have further demonstrated that both human ESCs and EMSCs, after aggregation to form spheroids, can tolerate hypoxic and ambient conditions (AC) for over 4 and 10 days, respectively, without loss of their viability and alteration of their functions. Based on these advantages, we decided to test whether EMSC spheroids, made in large quantity and delivered through a long-term distance at AC, can treat osteoarthritis spontaneously developed in rhesus macaques (*M. mulatta*) monkeys as well as the allogenic MSCs.

**Methods:** Xenogeneic AC-transported EMSC spheroids or allogenic BMSCs were injected into the articular cavity of both knees of the monkeys at 3 animals per group. Another two macaques were injected the same way with saline as controls.

**Results:** Both EMSCs and BMSCs groups showed significant amelioration indicated by the reduction of swelling joint size and amplification of keen flare angle post-treatment, compared to the control group. Examinations via X-ray and MRI also indicated the decrease of inflammation and osteophyma, and recovery of the synovium and cartilage in both treated groups. No sign of allergy or graft versus host disease was observed in the animals.

**Conclusion:** Our results demonstrate that human EMSC spheroids can prevent the osteoarthtitis progression and ameliorate osteoarthritis in the rhesus macaques as well as allogenic BMSCs, and this study shall help advance the clinical application of EMSCs.

## Introduction

Arthritis with bone/cartilage degradation and chronic inflammations can cause stiffness and chronic pain 1, which may develop into severe disability 2 and affect health and life quality of the patients 3. Osteoarthritis is the most prevalent degenerative disease in the elderly, who lose the capability of chondrocyte regeneration, endure cartilage degradation, are prone to bone fracture, experience variable inflammation and a high risk of muscle loss and even paralysis 4. Currently, there are no effective pharmaceutical drugs universally accepted for therapies of osteoarthritis, except for the purpose of short-term pain relief 5. Conventionally, physical treatments like lying down, reducing labor intensity, and hot water baths can relieve the pain at the early onset phase of osteoarthritis. However, these kinds of treatments are time consuming and may cause absenteeism for the work force 6. Additionally, some anti-inflammatory pharmaceutical drugs like acetaminophen can inhibit the severity of inflammation of osteoarthritis, but these are steroidal and hormonal drugs, which can induce severe addiction, endocrine turbulence, and obesity 7. The replacement with an artificial joint is the only option for patients at the later-stages of osteoarthritis. However, the surgery is invasive and painful, and the patients need a long-time for recovery with high risk of complications 8. Therefore, current therapies are unavailable to effectively restore cartilage regeneration.

In contrast, mesenchymal stem cell (MSC) based therapy has shown a great potency to regenerate cartilage and improve the symptoms of the disease with efficacy and safety 9. Intra-articular injections of bone marrow MSCs are efficacious for treatment of osteoarthritis in both rat and rabbit models 10, 11 as well as clinical trials^12^, ^13^. MSCs can respond to inflammatory cytokines *e.g.* IFN-gamma and IL-6, and secret anti-inflammatory factors such as IDO-2 and PD-1. Meanwhile, MSCs exert strong migration capability to the inflammatory location with increased expression of chemokines like CCL-2 and CXCR4 14. Those cytokines are encapsulated in cytoplasm and secreted into the micro-environment as exosomes to inhibit the inflammation and reduce the symptom of swelling and redness 15. MSCs can also differentiate into chondrocytes possibly participating in the rebuilding or recovery of cartilage 16. Previous reports indicated that MSCs can improve the symptoms of arthritis patients in clinical trials. No obvious side effects during MSC administration were observed after long-term observations 17, 18. It is putative that MSCs are safe and efficient for cell therapy in osteoarthritis 19-21.

Theoretically, autologous transplantation of MSCs is the ideal treatment of arthritis and many types of tissues can be the sources for MSC retrieval such as fat, bone marrow, gingiva, placenta, etc. 22. However, this strategy excludes patients with genetic deficiency or the elderly who yield MSCs with low capability in proliferation, multipotency, and therapeutic efficacy 23. Allogeneic transplantation of MSCs is an alternative option for the treatment of arthritis which facilitates pain relief and improves functions in movement in some licensed trials 24. Both autologous and allogeneic MSCs are derived from somatic tissue (st-MSCs), which have inevitable shortages due to their heterogeneity, rapid senescence, and slow propagation, *in vitro*. The tissue retrieval process is also invasive and causes pain to donors 25, 26. Pluripotent stem cells (PSCs) including embryonic stem cells (ESCs) and induced pluripotent stem cells (iPSC) have an unlimited self-renewal capability and can differentiate into any type of somatic cells including MSCs 27-29. MSCs derived from human pluripotent stem cells share similar characteristics with st-MSCs including positivity for typical cell surface markers, tri-lineage differentiation potentials, and immunomodulatory effects. MSCs derived from human embryonic stem cells (EMSCs) have proved to improve the symptoms in mouse disease models such as colitis, lupus, and experimental allergic encephalomyelitis (EAE) 14, 26, 30. Furthermore, EMSCs can be easily modified via genome editing and produced in large scale and at good manufacture procedure (GMP) grade, compared to st-MSCs 31-33. An increasing number of MSC production centers have been established to meet the high demands for basic and clinical studies. It is still challenging to control the MSC quality among various centers and hospitals 15, 34. Recently, the spheropreservation transporting MSC at ambient condition (AC) for more than 10 days retained the cell viability and therapeutic functions in animal studies which promote the shipping of stem cells to a remote site in a non-cryopreserved way^35^. Spheropreserved (AC-transported) EMSC spheroids alleviated the symptoms of mouse colitis and monkey EAE model^35^, ^36^.

Animal models play an important role in drug development and therapeutic studies on osteoarthritis 37. Currently, mouse 38 and rabbit 39 are the most widely used animals for drug intervention and toxicity studies on osteoarthritis. Compared to the lower species, rhesus macaque monkeys maintain an erect body and have biomechanical properties in their bones very similar to humans. Studies have shown that osteoarthritis begin to develop in adult rhesus macaques from the age of 5, which is structurally, chemically, and biomechanically similar to the case in humans 40. Therefore, nonhuman primates offer ideal animal models for human osteoarthritis research. In this study, macaques with spontaneous osteoarthritis were used to test the efficacy of AC-transported human EMSCs shperoids on this disease, with allogeneic bone marrow MSCs (BMSCs) used as a conventional control and saline as a negative control.

## Materials and methods

### Animals and ethic regulation

In a colony with over 1,000 rhesus macaques in Yunnan Key Laboratory of Primate Biomedical Research, some macaques were found to have developed osteoarthritis during annual physical examination. The macaques with swollen joints and partially lost movement capability were diagnosed to be at the early or middle stage of spontaneous osteoarthritis by 3 veterinarians and the swollen joints, inflammation, and formation of osteophyma were confirmed via X-ray and MRI exams. Eight of the osteoarthritis monkeys at the ages of 6-15 years were included in the study and divided into three groups. Groups 1 and 2 (with 3 monkeys/group) received intra-articular injection of human EMSC spheroids (EMSC_Sp_) and allogeneic BMSC, respectively. Group 3 (with 2 monkeys) received intra-articular injection of an equal amount of saline. Each animal was individually caged with the animal room set on a 12-h light and 12-h darkness cycle, the room temperature between 18 °C and 26 °C, and the humidity between 40% to 70%. The macaques were fed with monkey chow twice a day and supplemented with fresh fruits and vegetables once per day. All experimental procedures were reviewed and approved under protocol #LPBR20170201 by the Institutional Animal Care and Use Committee of Kunming University of Science and Technology and were performed in accordance with the Guide for the Care and Use of Laboratory Animals Eighth Edition. The veterinarian followed ARRIVE guidelines to ensure appropriate handling procedures for immobilization, sedation, and anesthesia. Abnormal postures, anorexia, vocalization, lethargy, and self-directed behaviors are indicators to evaluate pain or distress. New toys, cage changes, or comforting foods (such as peanuts, raisins or candies) are employed to moderate stress without compromising the scientific aspects of the experiments.

### Cells preparation and injection of macaque BMSCs and human EMSCs spheroids

BMSCs were isolated from the tibias of young rhesus macaques as described in our previous study 41. Briefly, the muscular tissues in the tibias were carefully removed and the ends of the tibias were cut on a benchtop. Bone marrow was aseptically flushed many times using a sterile syringe with Dulbecco's modified Eagle's medium (DMEM) (Gibco BRL, Grand Island, NY, USA) supplemented with 10% fetal bovine serum (FBS) (Gibco BRL, Grand Island, NY, USA) and 1% penicillin/streptomycin (Gibco BRL, Grand Island, NY, USA). Marrow cells were collected and mechanically dispersed into single-cell suspension after being centrifuged at 500 *g* for 5 minutes, and then seeded into a 10-cm plastic dish at a density of 1×10^6^ cells/ml. The cells were cultured in DMEM supplemented with 10% FBS at a 37 °C incubator with 5% CO_2_. The medium was refreshed every 48 hours. The primary cell culture (passage 0) was passaged with 0.25% Trypsin (Gibco BRL, Grand Island, NY, USA) after being cultured for about 10 days. The morphology, surface markers, and differentiation potency of BMSCs were identified at passage 3. The cells were collected at passage 4 following treatment with 0.05% Trypsin, then rinsed and re-suspended in 0.9% NaCl solution before articular injection.

The Envy hESC line constitutively expressing green fluorescent protein (GFP), maintained at University of Macau, was used in this study. The cells were induced to differentiate to MSCs via a 3D method as described in our previous report 42. Briefly, hESCs clusters were generated through a 50 μm strainer and treated with 10-ng/ml BMP4 and 1-μM A83-01 (Gibco BRL, Grand Island, NY, USA). After 3 days, the consumed medium was replaced with a MSC medium, which contained 20% FBS, 1% L-Glutamine and α-MEM basal medium (Gibco BRL, Grand Island, NY, USA). The medium was refreshed every 3 days until day 20 of the differentiation.

EMSCs were allowed to form spheroids as described previously 35. Briefly, EMSCs were dissociated to single cells at a density of 1 × 10^6^ cells/mL. Spheroids were formed from the dissociated cells in hanging drops with about 2.5 × 10^4^ cells/25 μL/drop in an incubator at 37 °C. The EMSC_Sp_ were harvested at 48 hours after start of the hanging drop culture, sealed in a 15-mL tube filled with the MSC medium, and then stored under AC for 3 days. The tubes containing MSC spheroids were transported to the animal facility in Kunming within 4 days. The spheropreserved EMSC_Sp_ at AC-D7 (EMSC_Sp-AC/D7_) were washed and re-suspended in 0.9% NaCl solution before the articular injection. As a conventional positive control, BMSC single cells suspension were prepared right before the cells treatments.

### Flow cytometry analysis for cell surface markers on MSCs

Expression of cell surface markers on MSCs was examined via flow cytometry using a commercial MSC Analysis Kit (BD Biosciences, San Jose, CA) according to the manufacturer's instructions, as described previously 41. Briefly, 5 × 10^5^ MSCs were collected, washed, and centrifuged in 500 μL of PBS supplemented with 3% FBS (PBSF). MSCs were then re-suspended in 100 μL of PBSF and incubated with antibodies on ice to detect cell surface markers including CD44, CD90, CD73, and CD105 and negative markers including CD45, CD34, CD11b, CD19, and HLA-DR. An isotype control antibody cocktail was used as a negative control. Unbound antibodies were washed away and the cells were re-suspended in 500 μL of PBSF, and examined via flow cytometry analysis.

### Evaluation of the differentiation potential of MSCs

MSC differentiation was conducted using the Tri-lineage Differentiation Kit (Gibco BRL, Grand Island, NY, USA) as described previously 43. For adipogenic differentiation, MSCs seeded in a 24-well plate at a density of 8 × 10^4^ cells per well were cultured in the adipogenic differentiation medium for 21 days with the consumed medium refreshed every three days. Derived adipocytes were stained red with Oil Red O (Sigma, St Louis, USA). For osteogenic differentiation, 4 × 10^4^ MSCs seeded in a 24-well plate were cultured in the osteogenic differentiation medium for 21 days with the consumed medium refreshed every three days. Derived osteocytes were stained positive for Alizarin Red. For chondrogenic differentiation, 2 × 10^5^ MSCs seeded in a 24-well plate were cultured with the chondrogenic differentiation medium for 21 days with the consumed medium refreshed every three days. Chondrocytes were stained positive with 1% toluidine blue (Sigma, St Louis, USA).

### Cell transplantation

Eight rhesus macaques with spontaneous osteoarthritis were randomly allocated into 3 groups for treatment with macaque BMSCs (n = 3), human EMSCs (n = 3), and normal saline (n = 2). For the BMSC group, 1 × 10^7^ allogenic BMSCs were collected and washed with normal saline 3 times, and the single cells suspended in 100-μL normal saline and injected into the joint cavity of both left and right knees (5 × 10^6^ cells/knee). For the EMSC group, EMSC_Sp-AC/D7_ containing 1 × 10^7^ cells were injected into the joint cavities of both knees (5 × 10^6^ cells/knee). For the normal saline group, the equal amount of saline solution was injected into the joint cavities as above. The MSC transplantation was performed monthly for 3 times.

### Measurement of the width of the knee joint

The width of the knee joint of each macaque was assessed three times using a Vernier caliper by two experienced veterinarians who were blinded in regard to macaque identity. For the width of arthrosis measurement, the knee joint of rhesus macaques was furthest and freely extended, keep vernier caliper vertical with the front of the knee joint when calculating, and the width of joint center is the result of measurement. It was measured seven times before cell transplantation and 1, 2, 3, 4, 6, and 9 months after the transplantation, respectively. For each knee joint, every measurement was repeated three times. To calculate of thickening join width, the width of knee joint of healthy macaque were measured corresponding to the age and weight of osteoarthritic macaque. The thickening degree of swelling of knee of each arthritic monkey before treatment was represented as 100%. The reduction of the thickening degree of knees after cell treatments of each arthritic monkey was shown respectively.

### Maximal extension degree of the knee

The knee joint extension degree refers to the angle between the tibia and femur of each macaque. Two blinded veterinarians measured it on both knees of each animal using a protractor after the hind limb was naturally stretched. The measurement was conducted seven times as above. For each knee joint, every measurement was repeated three times. To calculate the fold changes of extension degree, the extension degrees of each macaque on 1, 2, 3, 4, 6, and 9 months were normalized to extension degree of 0 month (before cells treatments).

### MRI and X-ray

The macaques were anesthetized and X-ray radiographs (WD-XD20A) of their bilateral knee joints were performed seven times as above. Each knee joint was photographed for its anterior, medial, and lateral positions. The MRI imaging was conducted on the 3T machine (Siemens). The MRI (3.0-T) scanning of the knees was performed on the macaques seven times as above. Articular cartilage was quantitatively assessed based on T1 rho and T2 relaxation times. Cartilage thickness and signal intensity of the surfaces of the patella, medial and lateral femoral, and tibial condyles were measured. Analysis of variance for random block design data, bonferroni test, and paired sample *t*-tests were performed to estimate the influences of physiological activities on articular cartilage.

### Data analysis

The statistical differences were analyzed using Student's* t*-test. and a *P* value less than 0.05 was considered significantly different. All of the data are presented as the means ± SD or SE. Line GraphPad Prism 8 was used for charts drawn and all calculations (GraphPad Software, San Diego, CA, USA).

## Results

### Preparation and functional verification of macaque BMSCs

**Figure 1:**
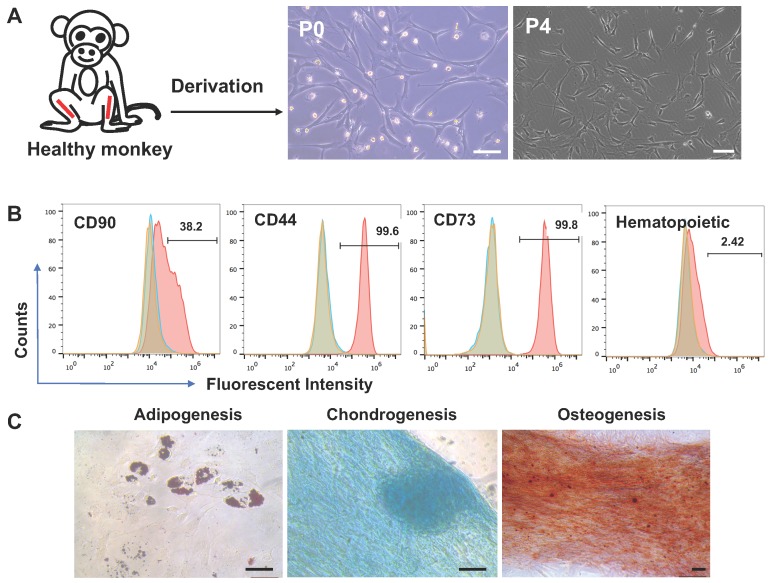
Characterization of BMSCs derived from a rhesus macaque. (A) Process of BMSCs derivation and morphology of the cells, Scale bars: 100 µm. (B) Flow cytometry assay for cell surface markers on the monkey BMSCs. Controls were set up as in Figure 1. (C) Tri-lineage differentiation of the monkey BMSCs. Scale bars: 50 µm.

Bone marrow cells were isolated from macaque tibias and plated in plastic dishes. The cells that formed colonies and appeared heterogeneous were referred to BMSCs in passage 0 (p0). The p0 cells were expanded through progressive subculture. By p3, the cells developed typical fibroblast-like morphology with elongated, spindle-shaped, heterogeneous, and single-nucleated structures (Figure 1A). Using flow cytometry analysis, the p3 cells were found positive at high ratios for the typical cell surface markers for MSCs including CD44, CD73, CD90, and CD105 but negative for the hematopoietic markers CD19, CD11b, CD31, CD45, and HLA-DR (Figure 1B). The cells also demonstrated capability to differentiate to adipocytes per Oil Red O staining, osteocytes per alizarin red staining, and chondrocytes per Alcian blue staining (Figure 1C).

### Functional verification of human EMSC spheroids following ambient transportation

**Figure 2:**
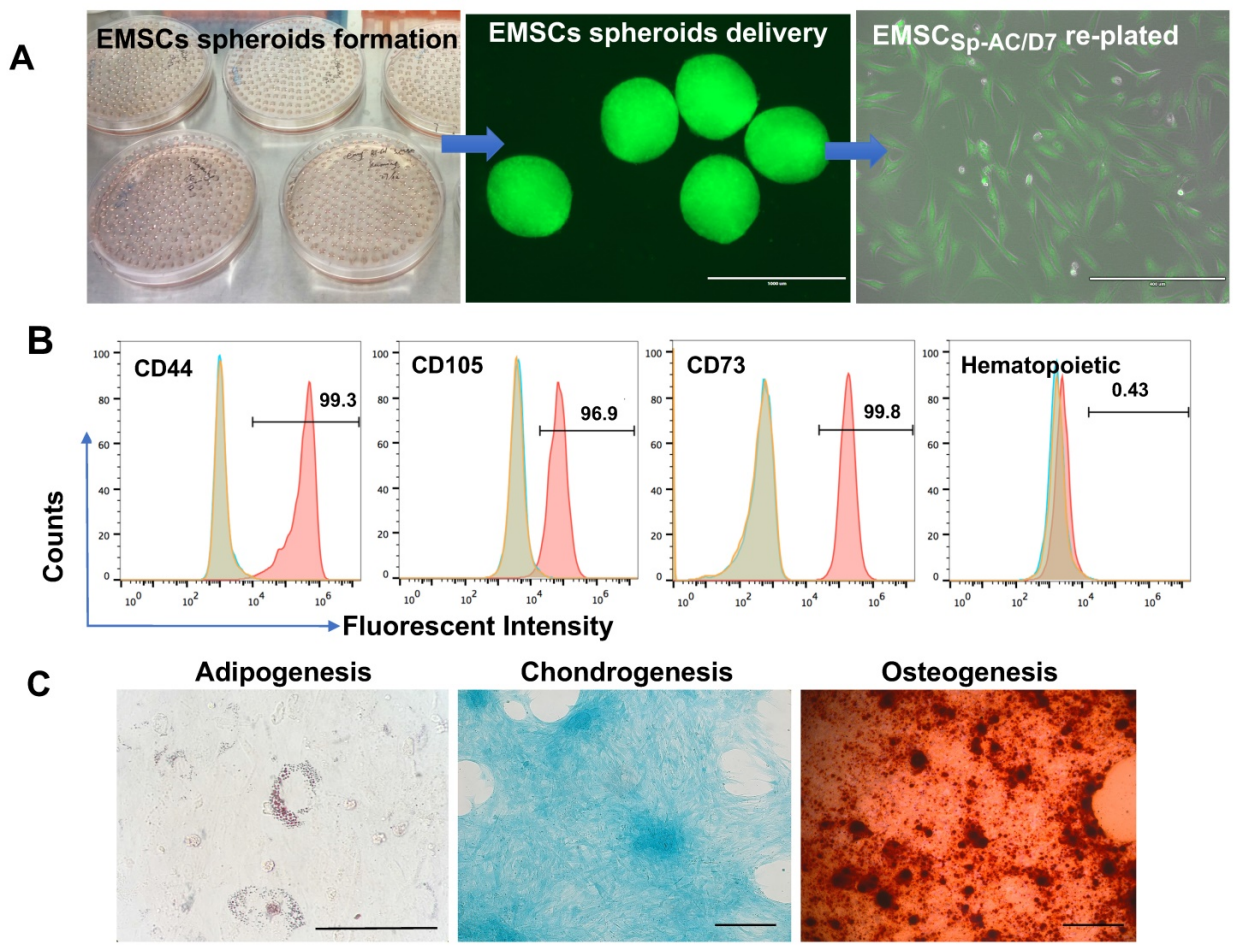
Characterization of EMSCs derived from the GFP+ Envy hESCs. (A) Formation of EMSC spheroids in hanging drops, morphology of the spheroids and EMSCs in monolayer after delivery and being re-plated in a Petri dish. Scale bars: 1mm (left) and 400 µm (right). (B) Flow cytometry assay for cell surface markers on EMSCs. The red peak refers to a tested sample, and blue and brown peaks refer to isotype and unstained controls, separately. (C) Tri-lineage differentiation of EMSCs into adipocytes, chondrocytes, and osteocytes. Scale bars: 100 µm.

EMSCs were derived from the Envy hESC line and EMSCs (Passage 8) were used to form spheroids in hanging drops as described 35, 44. The EMSC spheroids (EMSC_Sp_) were collected and transported in 15 ml tubes from Macau to Kunming (over 1,000 km) under AC for 7 days without medium refreshment and gas maintenance (Figure 2A and S1A). Upon arrival, EMSC spheroids at AC-D7 (EMSC_Sp-AC/D7_) were dissociated and the dissociated cells had 95% survival rate per AO/PI assay (Figure S1B). After being re-plated as a monolayer in 2D, the cells showed typical fibroblast-like morphology (Figure 2A). The EMSCs remained positive for typical MSCs markers CD44, CD73, CD90, and CD105 and negative for hematopoietic markers CD19, CD11b, CD31, CD45, and HLA-DR per flow cytometry analysis (Figure 2B). They retained the capability to differentiate to chondrocytes, osteocytes, and adipocytes (Figure 2C).

### EMSCs spheroids underwent the storage of ambient conditions directly differentiate into chondrocytes

**Figure 3:**
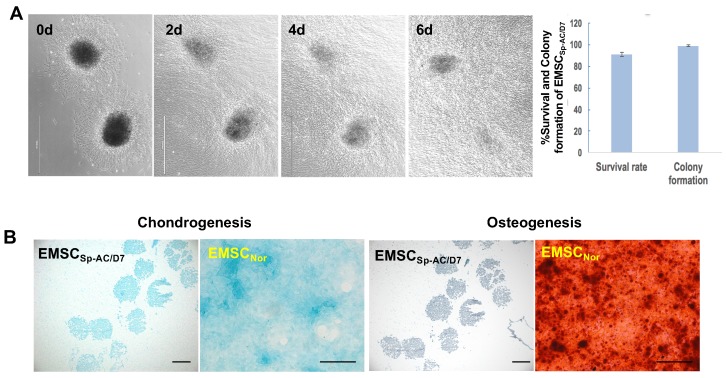
Viability and chondrogenic differentiation of EMSC spheroids post 7 days of ambient delivery. (A) EMSC spheroids underwent delivery at ambient condition for 7 days maintain high survival rate and fast recovery in dish after arrival in host facility. Scale bars: 1 mm. (B) EMSC spheroids delivered under ambient conditions for 7 days retained capability to directly differentiate to chondrocytes in 3D spheroids in chondrocyte differentiation medium, which were positive for the chondrocyte staining Alcian Blue but negative for the osteocyte staining Alizarin Red. EMSCs cultured in 2D (EMSCNor) were differentiated to chondrocytes and osteocytes in the corresponding medium as positive controls. Scale bars: 200 µm.

EMSC_Sp-AC/D7_ had 98% colony formation rate after being re-plated onto the 2D culture dish and migrated out cells with normal MSCs morphology on day 1 and throughout one week (Figure 3A). The EMSCs spheroids also held the capability to directly differentiate into the chondrocytes in the controlled differentiation medium and showed the cartilage specific verified by positive Alcian Blue staining and negative Alzarin Red staining, which indicates that the EMSCs spheroid can be sustained in the ambient conditions for 7 days and still hold the differentiation potential to cartilage (Figure 3B).

### EMSCs spheroids can sustain the hypoxia and less nutrient condition than dissociated cells

The EMSC_Sp-AC/D7_ were plated in the hypoxia incubator with 5% oxygen supply and less nutrients with 2% FBS in DMEM without glucose. The EMSCs spheroids well attached the surface of dish and migrate the cells overnight with normal MSCs phenotype but there were clear babbles in the cytoplasm of MSCs directly dissociated from the monolayer culture without spheroids formation and ambient storage (Figure S2A). The activated caspapse3 highly expressed in the dissociated MSCs control but few are positive in the EMSCs spheroids and its dissociated small clumps (Figure S2B). Those results indicated that the spheroid formation and ambient storage benefit the survival of EMSCs for transplantation in the hypoxia and less nutrient environment.

### EMSC spheroids reduce the knee joint and increase the extension degrees of the joints in osteoarthritis monkeys

**Figure 4:**
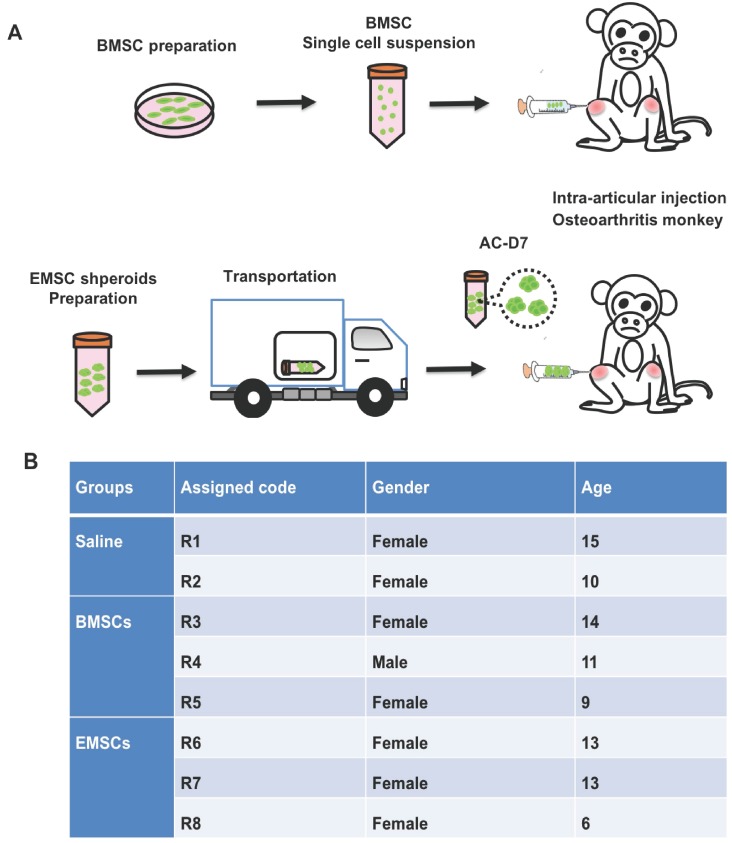
Treatment of osteoarthritis monkeys with MSC via intra-articular injection. (A) A scheme for BMSC preparation, ambient transportation of EMSC spheroids and intra-articular injection of the MSC into osteoarthritis. (B) The table of monkeys' information and the experimental treatments were shown below.

**Figure 5:**
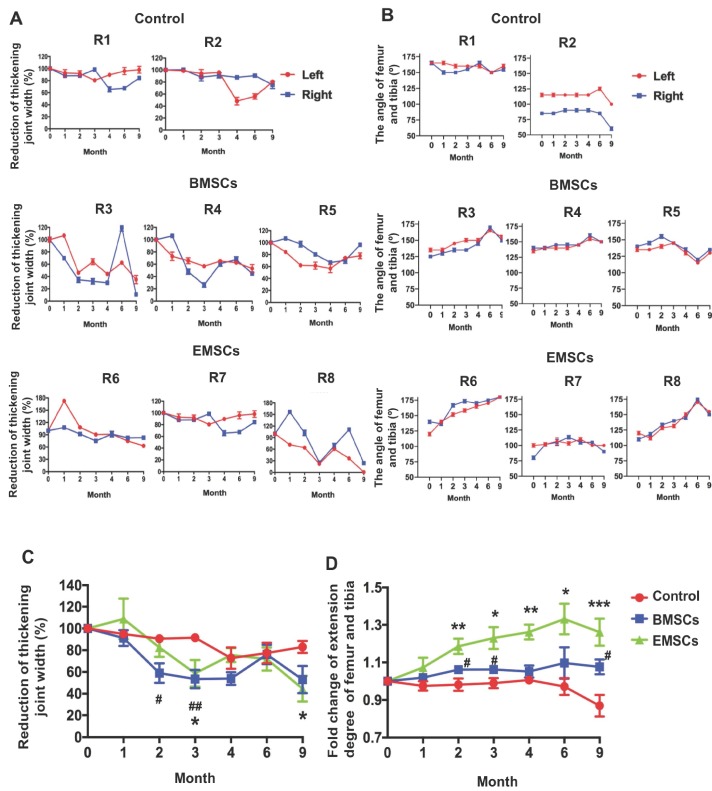
MSC-mediated amelioration of rhesus osteoarthritis in width of joint and leg extending. (A) The thickening joint width before treatment were described as 100%. The reduction of the thickening joint width were shown following the timeline of cell treatments. Data of three measurements by two experienced veterinarians on each knee are presented as means ± SD. (B) Timeline of the angle of femur and tibia. The macaques of the control group are R1 and R2. The macaques of the BMSCs treatment group are R3, R4 and R5. The macaques of the EMSCs treatment group are R6, R7, R8. Data are presented as means ± SD. (C) The statistics of the reducing joint width of three monkeys are presented as means ± SE. (D) The maximal extension degree of both knees measured as the angle formed by the femur and tibia of three monkeys. Data are presented as means ± SE. The red line graph represents the left knee and the blue line graph represents the right knee. The statistical differences were analyzed using Student's *t*-test. The significant differences of BMSCs and control groups are presented as #, P < 0.05; ##, P < 0.01. The significant differences of EMSCs and control groups are presented as *, P < 0.05; **, P < 0.01; ***, P <0.001.

Three monkeys with spontaneous osteoarthritis per group were treated with either EMSC_Sp-AC/D7_ or BMSCs via intra-articular injection. The monkeys of control group were injected with saline solution (Figure 4A and B). Both the width of the knee joint and extension degrees of the joints were measured before and after treatments with a Vernier caliper to determine the severity of swelling and recovery of arthritis. The thickening degree of the knees of each arthritic monkey before treatment was defined as 100%. As shown in Figure 5A and C, the thickening degree of knee joints decreased obviously in the osteoarthritis monkeys treated with either EMSCs or BMSCs during the first 3 month observations. After the rapid effect of the 3 injections of cell therapy, the improvement of reducing joint width progressed slowly and the rebound effects were observed during 4-6 months observation. However, the significant improvement were observed in both EMSCs and BMSCs treated groups from the 6 to 9 month. In contrast, the two macaques that received saline injections showed no decrease in the width of knee joints (Figure 5A and C), indicated that there was no obvious self-recovery of the knee joints in the control animals. On the other hand, the monkeys treated with either EMSCs or BMSCs gained improved extension degrees of knee joints on both hind legs during the continuous observation post-cell therapy (Figure 5B and D). Instead, the angles between the tibia and femur were decreased in the control group (Figure 5D). Moreover, the EMSCs treated group showed more significant improvement of extension degrees than BMSCs treated group, when compared to the control group. These results suggest that both EMSCs and BMSCs reduce the joint swelling caused by osteoarthritis and increase the flexibility of the joints.

### EMSC spheroids improves X-ray signs in osteoarthritis monkeys

**Figure 6:**
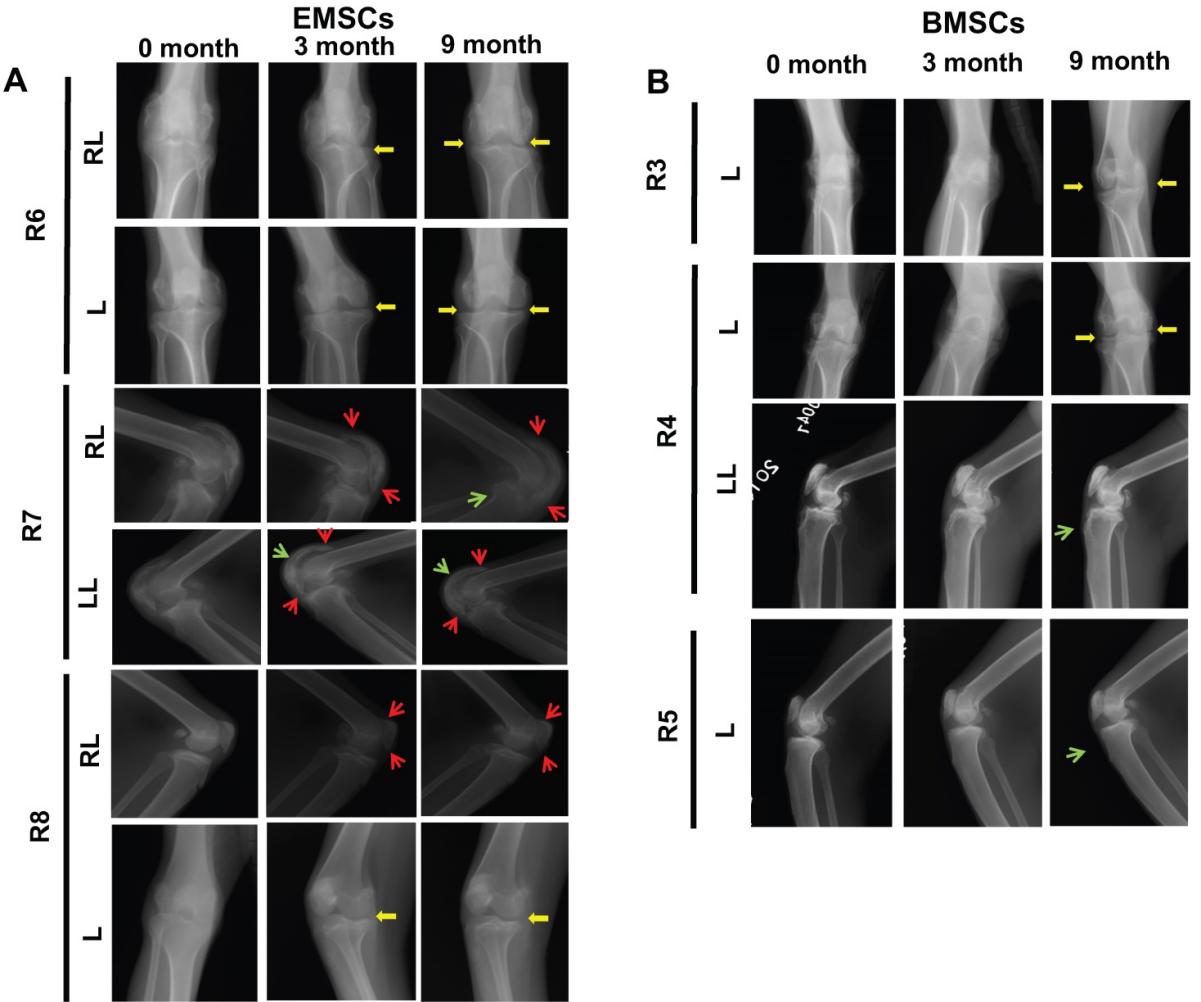
MSC-mediated amelioration of rhesus osteoarthritis per X-ray analysis on 3 month and 9 month. (A) X-ray imaging are shown for each monkey in EMSCs treated group. (B) X-ray imaging are shown for each monkey in BMSCs treated group. The bone hyperplasia (green arrow) and osteophyte formation (red arrow) were reduced. The gap of the joint (yellow arrow) increased for the knees of the monkey who received treatment with either EMSCs or BMSCs treatments. RL: Right lateral; LL: Left lateral; L: Left.

X-ray is a common tool for diagnosis of osteoarthritis and observation of joint changes during the disease course, which are often recorded according to Ahlback's criteria for osteogenesis 45. The whole knee joints of the macaques were imaged using X-ray to compare the changes before and after the MSC transplantation. Nine month post cells treatment, the X-ray images of both legs of the MSC-treated macaques (R3, R4, R6, and R8) showed obvious repair of the damaged knee joint space. All 6 monkeys with MSC treatments showed improvement of the morphology and density of the bones after the MSCs administration (Figure 6A and B). The shade of the joints, a sign of swelling, quickly reduced from the first three month after the MSC injection. The morphology of the joint bones became smoother and the osteophyma disappeared as pointed by the red arrow in Figure 6. In contrast, the two control macaques had aggravation of the above disease signs in their joints (Figure S3). Meanwhile, the arthritis gap was enlarged and the density of the joint bones improved from the third month to the ninth month in all three monkeys treated with EMSCs (Figure 6). These observations affirm both EMSCs and BMSCs prevent the progression and promote the recovery of osteoarthritis based on the X-ray records.

### EMSC spheroids improve MRI signs in osteoarthritis monkeys

MRI allowed the observations of the cartilage layer and synovial tissue of the animals with or without osteoarthritis. As shown in Figure S4, the MRI of the joints in the normal macaques shows a clear line of the bone growth plate, woven smooth and boneless ridges in the cartilage, and smooth and even synovial tissue in the joints.

In contrast, the rhesus macaque with osteoarthritis (R1) demonstrate obvious injury in the tibia end of the joints before injection of saline and unclear synovial tissue in MRI images (Figure S4). After three times of saline injection into the joint cavity, the distal femur injury remained in the animal, and the synovial tissue became even more uneven than before the injections. Obvious cartilage damage accompanied by bony outgrowth (osteophytes) was observed in the joints (Figure S4). Injuries in the cartilage layer were observed at the tibia and femoral ends before and three months after the saline treatment. Compared with the normal joints, the gap of joints remained big without obvious alteration after the saline treatment.

**Figure 7:**
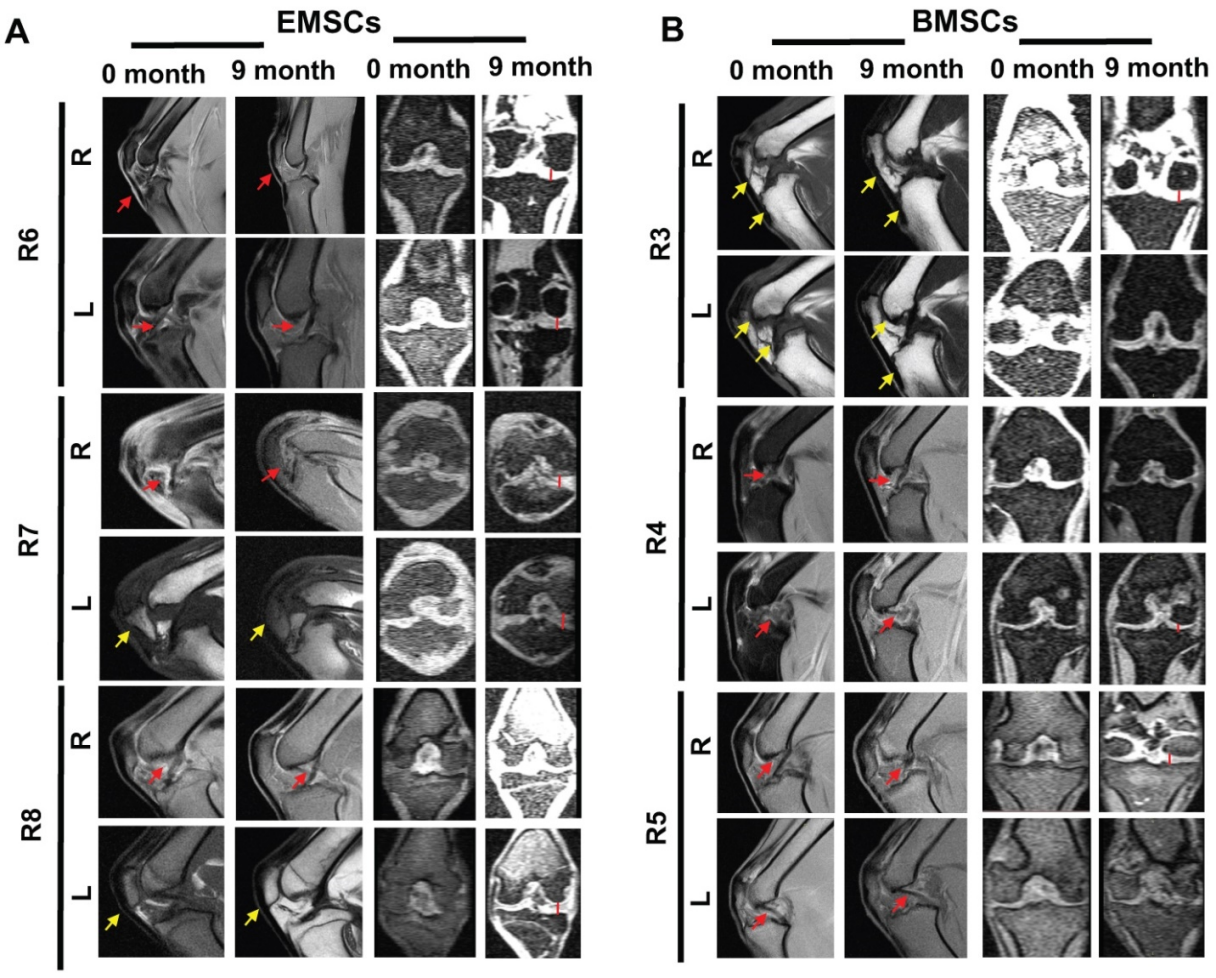
MSC-mediated amelioration of rhesus osteoarthritis per MRI analysis. The exudative patellar ligament (red arrows) decreased, bone hyperplasia (yellow arrows) decreased, and the gap of the joint (red straight line) increased for both knees of the monkeys 9 months after the treatment with EMSCs or BMSCs. R: Right; L: Left

On the other hand, improvement in osteoarthritis occurred in the groups treated with either dissociated BMSCs or EMSC spheroids injected into the joint cavity. Before the cell treatment, obvious injuries of cartilage and synovial tissue were observed at the humeral end. Although the synovial tissue still had hyperplasia one month after the cell transplantation compared to the pre-transplantation images, the hyperplastic synovial tissue subsided and the cartilage layer recovered in 9 months after the transplantation (Figure 7). In R4, the sectional MRI images showed obvious cartilage damage before the transplantation of BMSCs, and then improvement in the cartilage layer was observed after 3 consecutive transplantations of BMSCs (Figure 7B). In R6, obvious cartilage injury was also observed before and one month after injection of EMSC spheroids, and a distinct layer of cartilage was observed after 9 months of EMSC spheroids injection (Figure 7A). Increment of the gap of the joints, reduction of bone hyperplasia, and relief of exudative patellar ligament were observed in the monkeys after the transplantation of EMSCs or BMSCs. These results suggest that both EMSCs and BMSCs can improve syndromes and prevent deterioration in the osteoarthritis monkeys.

### No tumor formation in the joints injected with EMSC spheroids during 9-month observations

EMSCs derived from the Envy hESC line that constitutively expresses GFP 38 were used for checking tumor formation at various times following transplantation of the EMSC-formed spheroids. No tumor formation was identified in the joints through X-ray and MRI analyses, and no GFP DNA or RNA was detected in blood samples withdrawn from the animals 9 months after the transplantation (data not shown), indicating the biosafety of the human EMSCs administered in spheroids to the joints of the primates.

## Discussion

The non-human primate is the ideal animal species for modeling human diseases, exploring the therapeutic mechanism, and, most importantly, evaluating the efficacy and safety of new drugs and therapies aimed at clinical applications. The rhesus macaque is one of the most widely used non-human primates for generation of disease models. Macaques are similar to humans in kinship, walking style, the maintenance of an erect body, and the biomechanical properties of the bones including knee joints. Spontaneous osteoarthritis can develop in monkeys as well as humans. The present study has evaluated the efficacy of human EMSCs, in comparison with macaque BMSCs, on spontaneous osteoarthritis in macaques to advance the clinical applications of EMSCs.

MSCs derived from somatic tissues such as the bone marrow, fat, dental pulp, placenta, umbilical cord, and amniotic membrane are usually used for study of cell therapy. However, application of either allogenic or autogenic tissue-derived MSCs is restricted by the varying quality and limited number of donors and biosafety concern of pathogen transmission. On the other hand, the putative demand is high in clinic as a tremendous number of high-quality cells are often needed in a short time for cell therapy. It appears that EMSCs could overcome the hurdles as they are derived from unlimited hESC lines and easy for quality control and genetic manipulation if needed. In addition, it has been shown that, compared to BMSCs, EMSCs are faster in propagation, stronger against apoptosis, and slower in senescence 42. Like BMSCs, EMSCs have immune-modulatory and therapeutic effects in treatment of autoimmune diseases such as ulcerative colitis, uveitis, and multiple sclerosis 26, 36, 46. These properties overwhelm the disadvantages of tissue-derived MSCs, rendering EMSCs promising as a better source for MSC-based therapies. Recently, we reported that MSCs can be stored as spheroids at room temperature for up to 10 days without compromising their viability and biological functions, making it possible to deliver MSCs including EMSCs worldwide without need for cryopreservation 35.

It has been shown that tissue-derived MSCs are efficacious in treatment of osteoarthritis 47. However, the survival of transplanted cells is low in the joint cavity, as it has less amount of nutrients, lower oxygen, and more physical stress of movement than their source tissues such as the bone marrow or umbilical cord 48. It is critical for their therapeutic effects that the exogenous MSCs survive at least the first two days post-transplantation 49, 50. Enhanced anti-apoptosis capability has been observed with MSCs with increased Bcl-2 expression; pre-treatment with hypoxia environment can also increase the survival of MSCs via up-regulation of the *HIF* gene family 51,52. In both cases, MSCs are empowered to adapt to the transplanted environment with low levels of oxygen and nutrients.

MSCs can form spheroids when cultured in ultra-low attachment plates or hanging drops, which is similar to the condensation process of mesenchymal cells *in vivo*. In the spherical shape, MSCs form a tight shell in the outer-layer with high integrin expression and tight junction, however MSCs in the inner core have looser cell-cell contact, which helps MSCs adopt a hypoxic environment through increment expression of *HIF* and anti-inflammatory genes 36, 42. MSCs in spheroid culture can also tolerate hypothermic conditions as described above 35. In the articular cavity with arthritis, the space is so small that the bone ends touch closely causing friction of the cartilage surfaces. We speculate that MSC spheroids may survive better and longer than dissociated MSCs (which are widely used in studies of arthritis treatment) in the inflamed and stressed environment, thus able to achieve the therapeutic effects.

It appears the number of MSCs administrated to joints matters for the recovery of arthritis. Koga, *et al*. demonstrated that therapeutic effect is only present on cartilage defects when the transplanted MSCs reach the order of 10^7^ but not in the order of 10^6^ 53. However, Agung, *et al*. pointed out that administration of 10^7^ MSCs causes synovial complications in the joint cavity 54, and MSCs given at 10^6^/mL or less also led to significant therapeutic effects 55. These discrepancies may be associated to different qualities of the MSCs, different severities of the cartilage damage, and different animal models used.

In the present study, 10^7^ MSCs per animal was injected into the two joint cavity 3 times at an interval of one month to ensure that a sufficient number of MSCs be retained in enough time to act on the damaged joints. It has been reported that MSCs can adhere to the surface of articular cartilage by injection into the joint cavity and can differentiate to chondrocytes to repair damaged cartilage. It has been known that MSCs participate in tissue repair mainly by regulating the microenvironment of the damaged tissues 56. The immunomodulatory and anti-inflammatory effects of MSCs play a pivotal role in the treatment of osteoarthritis by regulating the maturation of T lymphocytes, reducing the levels of pro-inflammatory factors such as TNFα and interferon-γ, and increasing the levels of anti-inflammatory factors such as IL-4 and IL-10 14, 57.

## Conclusion

In summary, this study has demonstrated that AC transported EMSCs, like BMSCs, were efficacious for spontaneous osteoarthritis in rhesus macaques. The cells were injected in spheroids into the joint cavity of both knees of the animal and the animals were observed for nine months. The articular swelling was reduced and the extension degree in knee joint increased. Consistent with imaging tracking via X-ray and MRI, MSCs treatments prevent the osteoarthritis progression and promote the recovery of damaged joint space, bone hyperplasia, and exudative patellar ligament. Although osteoarthritis monkeys did not recover to the healthy condition as well as normal control and no mechanistic studies were conducted to elucidate how MSC spheroids execute the therapeutic effects due to the limited number of the monkeys and high cost of such projects, this study provides valuable information to advance the therapeutic application of EMSCs and spheroids transportation to patients.

## Supplementary Material

Supplementary figures and tables.

## Abbreviations

MSC: mesenchymal stem cells
EMSCs: MSCs differentiated from human embryonic stem cells
AC: ambient conditions
BMSCs: bone marrow MSCs
st-MSCs: MSCs derived from somatic tissue
PSCs: pluripotent stem cells
ESCs: embryonic stem cells
iPSCs: induced pluripotent stem cells
EMSC_Sp_: EMSC spheroids
EMSC_Sp-AC_: spheropreserved EMSC_Sp_ at AC
MRI: magnetic resonance imaging
