# Supplementary material for: Transplantation of human ESC-derived mesenchymal stem cell spheroids ameliorates spontaneous osteoarthritis in rhesus macaques
Source: Theranostics. 2019 Aug 21;9(22):6587–600. doi: 10.7150/thno.35391 (PMC6771254; doi:10.7150/thno.35391)
Supplement: Supplementary file 1 — Supplementary figures and tables. [file thnov09p6587s1.pdf]

Supplemental file:

Transplantation of human ESC-derived mesenchymal stem cell spheroids ameliorates spontaneous osteoarthritis in rhesus macaques

Bin Jiang [1, 2*], Xufeng Fu [1, 3*], Li Yan [2*], Shanshan Li [1*], Dongliang Zhao [4], Xiaoyan Wang[2], Yanchao Duan[1], Yaping Yan [1], Eqnin Li[2], Kunhua Wu [5], Briauna Marie Inglis [1], Weizhi Ji [1**], Ren-He Xu [2**], Wei Si [1**]

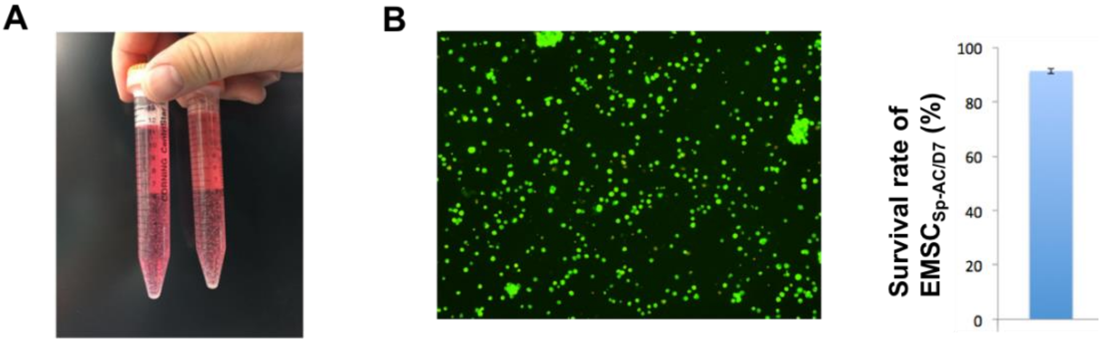

Figure S1. EMSC$_{Sp-AC/D7}$ spheroids holds high survival rate at arrival in host facility.

(A) EMSCs spheroids were sealed in the tube filled with medium for delivery (B) EMSCs underwent delivery at ambient condition for 7 days and dissociated for survival test with Live/Dead assay. Live cells: green and dead cells: red. EMSC$_{Sp-AC/D7}$ maintain over 90% survival rate at arrival in host facility for application.

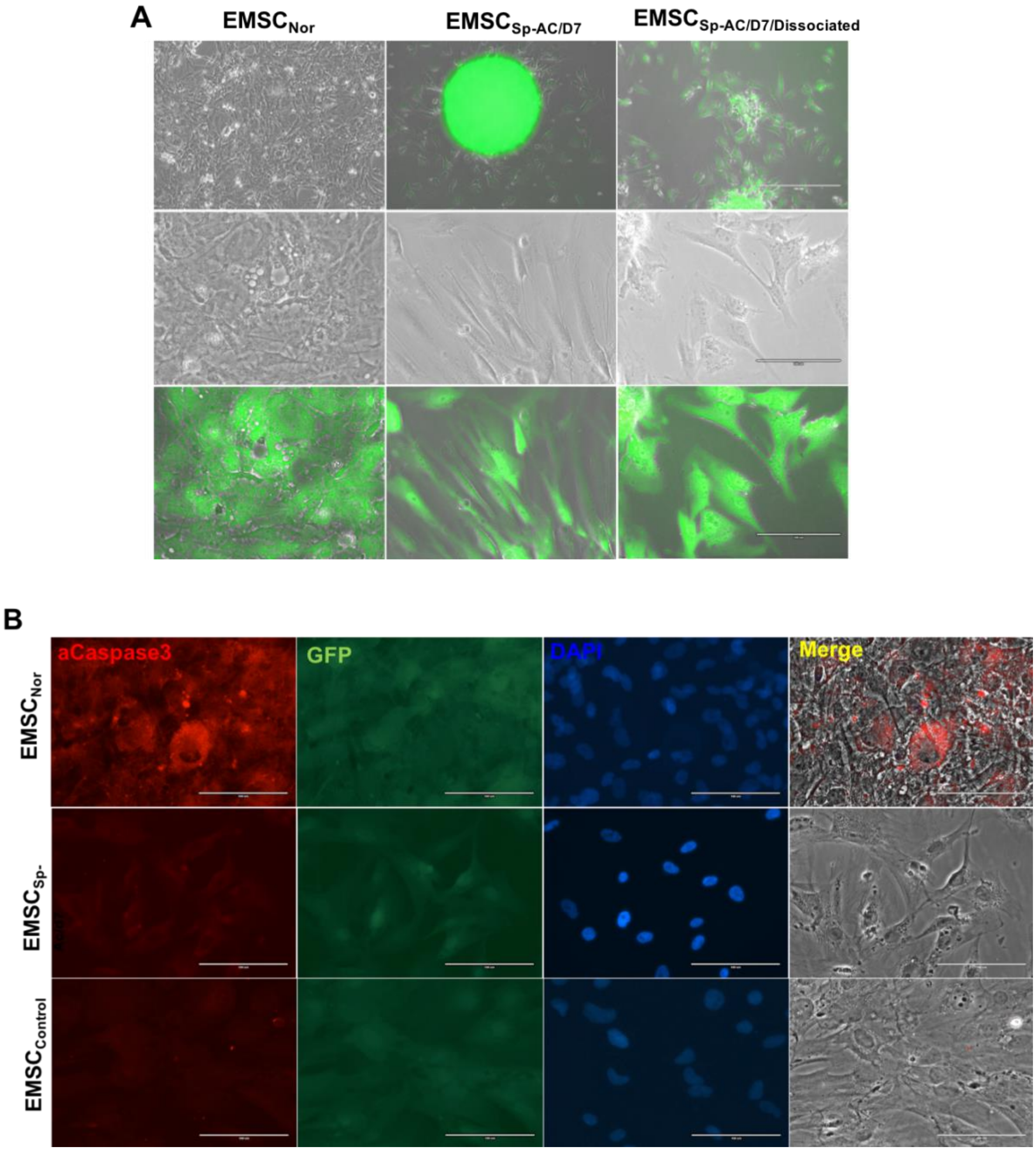

Figure S2. EMSC and BM-MSC spheroids benefits the survival more than single cell inoculation. (A) Morphology of EMSC$_{Nor}$, EMSC$_{Sp-AC/d7}$, and EMSC$_{Sp-AC/d7/Dissociated}$ cultivated in hypoxia and less nutrient condition at density of 1×10^6 cells/ml. Obvious babbles presented in the EMSC$_{Nor}$ but normal fibroblasts like cells in EMSC$_{Sp-AC/d7}$, and EMSC$_{Sp-AC/d7/Dissociated}$ samples. Scale bars: 100µm. (B) EMSC spheroids benefit the survival more than single cell inoculation. EMSCs released from spheroids underwent delivery at ambient condition for 7 days with less apoptosis cells which positive for activated caspase 3 than dissociated single cells from the monolayer control with the same density in inoculation under hypoxia and less nutrient condition. Scale bars: 100µm.

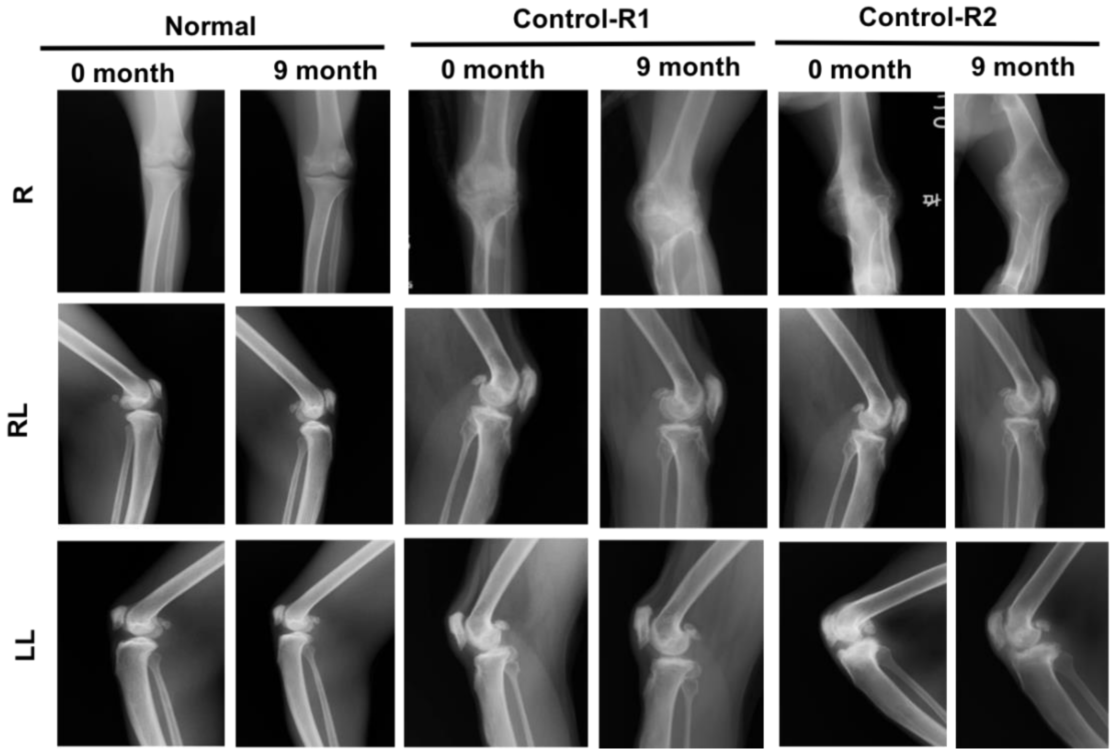

Figure S3. X-ray imaging of the knee joints for the normal monkey and two untreated osteoarthritis monkeys (R1 and R2) which had aggravated swelling and bone hyperplasia. R: Right; RL: Right lateral; LL: Left lateral.

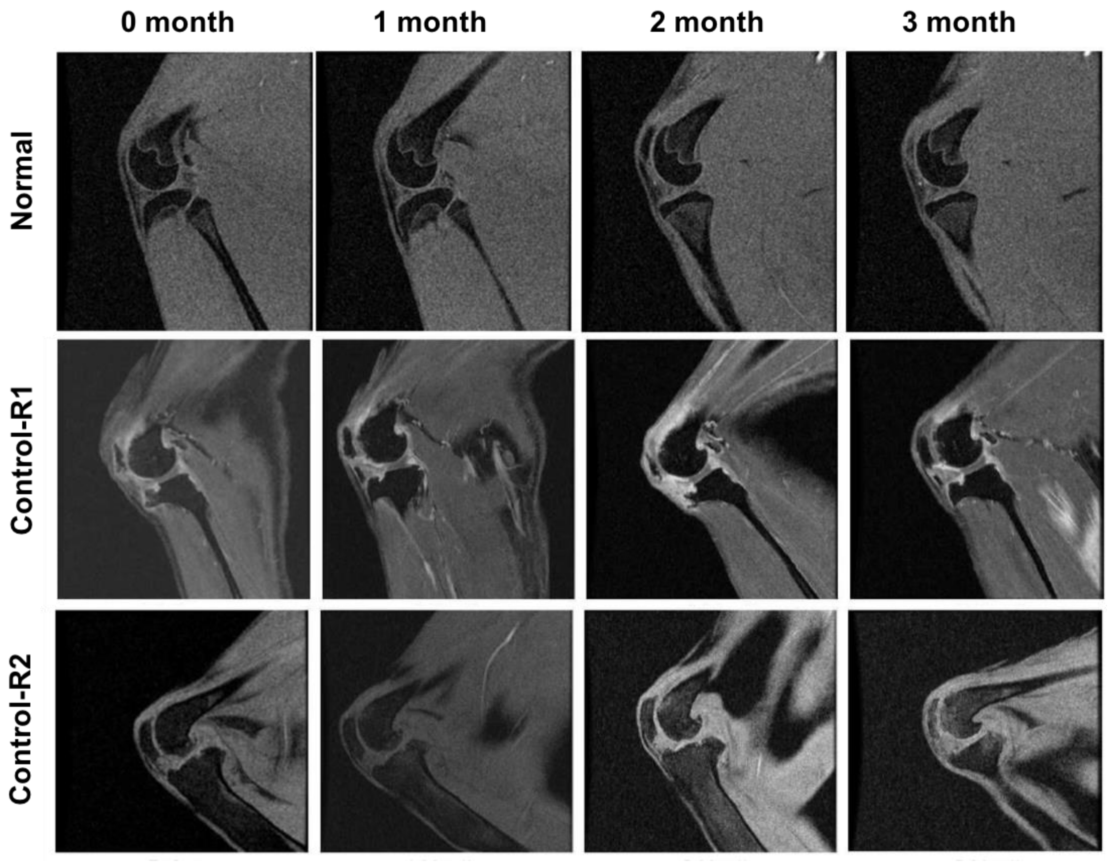

Figure S4. MRI imaging of the knee joints for the normal monkey and two untreated osteoarthritis monkeys (R1 and R2).
